# Study on the adaptability of multilayer subway network under sudden large passenger flow disturbances

**DOI:** 10.1371/journal.pone.0350567

**Published:** 2026-06-12

**Authors:** Nan-nan Lin, Hua-wei Yuan, Hong-tao Huang, Zhi-gang Liu

**Affiliations:** School of Urban Rail Transit, Shanghai University of Engineering Science, Shanghai, China; School of Systems Science, Beijing Jiaotong University, CHINA

## Abstract

This paper proposes a multilayer network-based subway network adaptability assessment framework.Adaptability is defined as the ratio of post-disturbance cumulative network performance to its nominal value, represented as 0≤ $F^{*}$≤1. In this framework, each line is treated as a separate layer, with the introduction of composite edge weight and platform congestion factor, and the establishment a performance response function. By integrating the “physical accessibility-perceived impedance” flow diversion model and bond percolation theory, the framework characterizes failure propagation, and is verified using the Shanghai Metro regional network, with fair comparisons made against the weighted single-layer network (WSN) and the multigraph model (MLG). The results show that the critical threshold of the multilayer network is 12% and 11% higher than that of WSN and MLG, respectively; under severe attack, the network performance exceeds $F^{*}$ by 23%−33%, and *ΔK* is 22%−26% lower. Monte Carlo variance analysis further indicates a significant interaction (p < 0.01) between passenger flow arrival and route choice.

## 1. Introduction

The rapid expansion of the Shanghai Metro network has introduced significant passenger flow challenges, characterized by extensive spatial distribution and peak-period aggregation. However, conventional static scheduling models are increasingly revealing limitations, primarily manifesting as significant response delays and insufficient accuracy. Because these models rely on fixed timetables and preset plans, they struggle to adjust train headways or dispatch additional services dynamically during sudden surges in passenger demand. Consequently, passengers often face extended delays, and the resulting bias between scheduled plans and actual operational status degrades both transportation efficiency and service quality.

Addressing the above problems, scholars have already conducted relevant research from multiple dimensions. In 1948, Norbert Wiener [1] first proposed Cybernetics, emphasizing the ability of systems to achieve Self-regulation through Feedback Mechanism, which laid the methodological foundation for subsequent Adaptive Network research; in 2002, Motter and Lai [2] established the “Load-capacity” framework for Complex Network Cascading Failure and designed the Load Balancing Adjustment Mechanism, providing systematic analytical tools for subsequent research; in 2004, Wu Jun and Tan Yueji [3], addressing the characteristics of Complex Network, first proposed a new measure for Complex Network connectivity—the Connectivity Coefficient; they conducted Network Invulnerability analysis using the world trade network as an example.Currently, research on Traffic Network Adaptability primarily revolves around three aspects: traffic network model construction, Resilience Assessment, and Cascading Failure Analysis. However, critical research gaps remain in these areas. In the `realm` of model construction, various Single-mode Traffic Network Model [4], and Multi-modal Traffic Network Mode [5,6] have been developed, yet existing research mostly focuses on the network’s Physical Structure, with insufficient attention to the differences in operational characteristics (such as Operating Speed, Departure Interval, and Dwell Time). Even though some studies have established a “Bus-Metro” multi-modal network model and incorporated factors such as Departure Interval, Passenger Capacity, and inter-station speed [7], there is still a lack of in-depth exploration into the differences in Operational Characteristics of different metro lines and the heterogeneity of transfer ‘channels’. From the perspective of Resilience Assessment, common metrics include network efficiency [8], average shortpath length, node centrality, and maximum connected subgraph [9], among others. However, most of these metrics fail to fully consider key factors such as time loss during passenger travel, making it difficult to comprehensively reflect the comprehensive response ability of transportation networks during disturbance events. In terms of Cascading Failure research, existing studies mainly focus on attack strategies and failure propagation models, for example, Li Guoliang et al. embedded a coupled map lattice model into organization networks [10], and Ma Yaping et al. coupled the MIKE 21 hydrodynamic model into road networks [11]; Inspired by the above research, this paper supplements the failure model with nondimensional analysis and conducts similarity analysis to enhance the model’s generalizability, achieving cross-scale resilience diagnosis.

To further clarify the contributions of this study, Table 1 provides a systematic comparison between the proposed multilayer framework and existing research across multiple dimensions.

**Table 1:** Comparison of the proposed framework with existing studies.

Dimension	Existing Studies	This Study
Research Question	Focus on physical topology and static connectivity.	Integration of physical accessibility and dynamic operational characteristics.
Model Structure	Single-layer or multigraph with “super-nodes”.	“Line-as-layer” multilayer configuration with inter-layer transfer edges.
Algorithm	Adjacent reassignment or shortest path assumptions.	“Physical accessibility-perceived impedance” diversion model.
Criticality Analysis	Degree centrality or static resilience metrics.	Nonlinear failure propagation based on bond percolation and $F_r$.
Operational Detail	Uniform capacity and ignoring dwell time.	Composite weighting including travel and dwell time.

To bridge these gaps, this paper proposes an improved Multilayer Subway Network self-adapting research framework. The framework independently characterizes each line’s Operational Characteristics based on the ‘line as a layer’ concept, enabling the dynamic migration of passenger flow in multi-layer space through inter-layer transfer edges. By introducing a performance response function and a ‘physical accessibility-perceived impedance’ flow diversion model, the proposed method overrides the traditional adjacent reassignment assumption. Finally, the study combines bond percolation theory with the crowding Froude number to achieve cross-scale resilience diagnosis. Taking the Shanghai Metro regional network as an example, the model’s effectiveness is verified in a fair comparison with the weighted single-layer network (WSN) and the multigraph model (MLG).

The remainder of this paper is organized as follows: Problem description and multilayer subway network attributes analysis introduces the construction of the multilayer subway network model and its improved weighting calculation method. Establishment of an improved adaptive evaluation model for multilayer metro networks presents the adaptive evaluation model, including the performance response function. Construction of an improved chain failure model for multi-layer metro networks elaborates the improved chain failure model and conducts scale similarity analysis. Case study analysis provides the simulation results and discusses the critical suppression mechanism. Conclusions summarize the whole work.

## 2. Problem description and multilayer subway network attributes analysis

### 2.1. Multilayer subway network model construction

Traditional single-layer networks abstract the subway system into a homogeneous “node-edge” topology. Its limitation lies in flattening the heterogeneity of lines, simply treating lines with varying capacities, speeds, and signal systems as homogeneous edges; the coupling of multiple lines within transfer stations often relies on “super-nodes”, leading to node degree bloat and path length aliasing. Additionally, the line load can only circulate along a single logical layer, making it impossible to characterize the dynamic migration of passenger flow in the multilayer spatial structure of “carriages—station halls—transfer channels”.

To overcome the above bottlenecks, this paper constructs a more realistic multilayer Subway Network model. Centered on the core idea of ‘line as layer’, this model maps each subway line with differing Operational Characteristics to an unaffiliated network. Transfer stations are no longer merged into a single point, but instead retain independent replicas in different layers, forming a multi-layered representation of ‘physically the same station, logically different layers’. Layers are coupled through a collection of transfer edges, enabling cross-layer passenger flow migration. Compared with traditional single-layer networks, the multilayer network preserves the operational variations of individual lines while providing standby paths. In the event of a node failure, passenger flow can be immediately redirected to other layers through inter-layer edges, enabling a cross-layer dynamic load redistribution mechanism that is not supported by single-layer or weighted graph models. A quantitative comparison showing the multilayer network’s accuracy is provided in Table 2(data comparison based on the Shanghai regional network in Section 4.1).

**Table 2:** Quantitative Comparison of Multi-layer, Single-layer and Multi-graph Models.

Modeling dimensions	Single-layer Network	Multi-graph	Multilayer Network in This Paper
Node Heterogeneity	Does not support	Partially supports	Fully supports (Independent parameters)
	Ⅱ	5.9-8.3	中风险
Transfer Station Representation	Single-node expansion	Degree distortion	Logical layering, degree preservation
Path Length Error	+22.7%	+9.4%	Benchmark
Flow Redistribution Error	+31.0%	+14.0%	Benchmark

Note: The errors are normalized with the results of the multi-layer network as the standard.

In the Multilayer Subway Network model, the collection of network layers is defined as *G*, and the collection of transfer edges between different network layers is denoted as *C*. Additionally, the adjacency matrix is represented by *A*, and the edge weight matrix is represented by *W.* In the *α*-layer network, the collection of nodes is denoted as *V*_*α*_, and the collection of directed edges is denoted as *E*_*α*_. Specifically, the collection of transfer edges between the *α*-layer and *β*-layer networks is defined as *C*_*αβ*_. In the adjacency matrix A of the Multilayer Subway Network, the adjacency matrices corresponding to the directed edges in the α-layer and *β*-layer are denoted as *A*^[*α*]^ and *A*^[*β*]^, respectively, while the adjacency matrix corresponding to the connecting edges between the *α*-layer and *β*-layer is denoted as A *A*^[*αβ*]^. If there exists a connecting edge between node *i* and node *j* in the *α*-layer network, the corresponding element in the adjacency matrix takes the value 1; otherwise, it is 0.Similarly, if there exists a connecting edge between node *i* in the *α*-tier network and node *j* in the *β*-tier network, the corresponding element in the adjacency matrix is assigned a value of 1; otherwise, it is 0.

### 2.2. Fundamental assumptions

To ensure the feasibility and scientific rigor of the multilayer network model and the subsequent cascading failure simulation, the following fundamental assumptions are established based on the Code for Design of Urban Rail Transit Stations and operational realities:

(1) Constant Vehicle and Line Capacity: It is assumed that the passenger capacity of trains and the transport frequency of lines remain constant during the sudden passenger flow disturbance. This allows the model to focus on the congestion dynamics at the station platform level rather than microscopic train-to-train variations.
(2) Homogeneous Platform Effective Area: The platform area available for passengers at each station is assumed to be homogeneous relative to its designed capacity class. This simplification is necessary for calculating a standardized platform crowding degree *K*_*j*_(*t*) across diverse station architectures.
(3) Rational Passenger Rerouting: Passengers are assumed to be rational agents who reselect their travel routes based on the dual criteria of “physical accessibility” and “perceived impedance”. This overrides the assumption of random movement and aligns with the nearest-transfer principle observed in actual urban rail transit systems.
(4) Uniform Passenger Reaction and Walking Speed: While individual walking speeds vary, the model adopts a calibrated average walking speed *v*_walk_ for all passengers within the transfer corridors to represent the collective movement characteristics during peak hours.
(5) Simplified Environmental Impact: External factors such as weather conditions or non-structural station maintenance are assumed to have negligible effects on the fundamental network topology and basic walking impedances during the evaluation period.

### 2.3. Improved network weighting calculation

The impact of Sudden Large Passenger Flow on the city subway network is more accurately represented by a composite weighting method that incorporates both passenger travel time and platform dwell time as edge weights. The calculation of edge weights between two adjacent nodes is given by equations (1) and (2).


$$\begin{array}{c} \overset{\alpha }{\underset{i^{\alpha }j^{\alpha }}{w}}=\overset{\alpha }{\underset{i^{\alpha }j^{\alpha }}{r}}+\overset{\alpha }{\underset{i^{\alpha }j^{\alpha }}{z}} \end{array}$$ (1)

$$\begin{array}{c} \overset{\alpha \beta }{\underset{i^{\alpha }j^{\beta }}{w}}=\overset{\alpha \beta }{\underset{i^{\alpha }j^{\beta }}{r}}+\overset{\alpha \beta }{\underset{i^{\alpha }j^{\beta }}{z}} \end{array}$$ (2)

In the equation, the edge weight between two adjacent nodes within the α-layer network is denoted as $\overset{\alpha }{\underset{i\alpha j\alpha }{w}}$, while $\overset{\alpha \beta }{\underset{i\alpha j\alpha }{w}}$ represents the edge weight between adjacent nodes across the α-layer and β-layer networks. The travel time between station *i*^*α*^ and station *j*^*α*^ in the α-layer network, corresponding to the actual operating time of the subway train between these two stations, is represented by $\overset{\alpha }{\underset{i\alpha j\alpha }{r}}$. The travel time between station *i*^*α*^ in the α-layer network and station *j*^*β*^ in the β-layer network, referring to the walking (transfer) time for passengers, is denoted as $\overset{\alpha \beta }{\underset{i\alpha j\alpha }{r}}$. $\overset{\alpha }{\underset{i\alpha j\alpha }{z}}$ and $\overset{\alpha \beta }{\underset{i\alpha j\alpha }{z}}$ indicate the dwell time resulting from Sudden Large Passenger Flow, during which passengers experience waiting or delayed boarding due to train car capacity constraints after the subway vehicle arrives at station *j*^*α*^ and station *j*^*β*^ from station *i*^*α*^.

## 3. Establishment of an improved adaptive evaluation model for multilayer metro networks

### 3.1. Construction of performance response function for multilayer metro networks

First, by comprehensively considering passenger travel efficiency and network service quality, a network performance response function [12], denoted as *F*_(t)_, is introduced to capture the instantaneous status of the network. The expression of the performance response function *F*_(t)_ at any given time *t* is:


$$\begin{array}{c} F(t)=\eta P+(\text{1}-\eta )D \end{array}$$ (3)

In the formula: *P* and *D* represent the passenger travel efficiency ratio and the network service efficiency ratio, respectively; *η* denotes the weight coefficient of the passenger travel efficiency ratio, and 1-*η* corresponds to the weight coefficient of the network service efficiency ratio.

The passenger travel efficiency ratio *P* reflects the change in passenger travel convenience after station failure, as shown in Equations (4)-(6):


$$\begin{array}{c} P=\frac{P^{**}}{P^{*}}=\left[\frac{\text{1}}{N^{*}\left(N^{*}-\text{1}\right)}\sum_{i,j\in V^{*},i=\;\;/\;j}\frac{\overset{*}{\underset{ij}{f}}}{\overset{*}{\underset{ij}{t}}}\right]/\left[\frac{\text{1}}{N\left(N-\text{1}\right)}\sum_{i,j\in V,i=\;\;/\;j}\frac{f_{ij}}{t_{ij}}\right] \end{array}$$ (4)

$$\begin{array}{c} t_{ij}=\overset{\alpha }{\underset{i^{\alpha }j^{\alpha }}{r}}+\overset{\alpha }{\underset{i^{\alpha }j^{\alpha }}{z}} \end{array}$$ (5)

$$\begin{array}{c} t_{ij}=\overset{\alpha \beta }{\underset{i^{\alpha }i^{\beta }}{r}}+\overset{\alpha \beta }{\underset{i^{\alpha }i^{\beta }}{z}}+\overset{\beta }{\underset{i^{\beta }j^{\beta }}{r}}+\overset{\beta }{\underset{i^{\beta }j^{\beta }}{z}} \end{array}$$ (6)

In the formula: P^*^ is the passenger travel efficiency weighted by the passenger flow of the original network, and P^**^ is the passenger travel efficiency weighted by the passenger flow after partial stations fail; *N* and N^*^ are the number of nodes in the original network and the damaged network, respectively; *V* and V^*^ represent the sets of nodes in the original network and the damaged network, respectively; *f*_*ij*_ is the passenger flow from station *i* to station *j*, and *f*_ij_^*^ is the passenger flow from station *i* to station *j* after partial stations fail; *t*_*ij*_ is the time between station *i* and station *j*, and *t*_ij_^*^ is the time between station *i* and station *j* after partial stations fail. Among them, in Equation (5), *i* and *j* are in the same layer, and in Equation (6), *i* and *j* are not in the same layer.

The network service efficiency ratio D reflects the change in network service efficiency after station failure. To better reflect the passenger detention experience, a platform congestion decay coefficient *γ* is introduced, as shown in Equation (7).


$$\begin{array}{c} D=\frac{D^{**}}{D^{*}}\times \frac{\text{1}}{\text{1}+\gamma \cdot \Delta K}=\frac{\sum_{i,j\in V^{*}i=\;\;/\;j}\overset{*}{\underset{ij}{\theta }}\overset{*}{\underset{ij}{f}}}{\sum_{i,j\in V\;i=\;\;/\;j}\theta_{ij}f_{ij}}\times \frac{\text{1}}{\text{1}+\gamma \cdot \Delta K} \end{array}$$ (7)

$$\begin{array}{c} K_{j}(t)=\frac{Q_{j}\left(t\right)}{C_{j}},\;\Delta K=\overset{*}{\underset{j}{K}}-\overset{**}{\underset{j}{K}}, \end{array}$$ (8)

In the formula: D^*^ is the total passenger flow of the original network, and D^**^ is the total passenger flow unaffected by travel after partial stations fail; *γ* is the congestion decay coefficient; the platform congestion degree *K*_*j*_(*t*) is determined by the load *Q*_*j*_(*t*) and the maximum capacity *C*_*j*_ of station *j*, and *∆K* is the change in platform congestion degree in the neighborhood of failed stations, as shown in Equation (8); *K*_j_^*^ and *K*_j_^**^ are the platform congestion degrees of station *j* before and after the failure, respectively; both *θ*_*ij*_ and *θ*_ij_^*^ are 0–1 variables, representing the connection relationship between stations *i* and *j* in the original network and the damaged network, respectively, taking 1 when there is a connection between stations *i* and *j*, and 0 otherwise.

### 3.2. Adaptive evaluation model

As defined in this study, adaptability is the ratio of the cumulative performance of the multilayer metro network after sudden large passenger flow disturbances to its performance under normal conditions. This metric can be measured by the following formula, as shown in Equation (9).


$$\begin{array}{c} F^{*}(t,e)=\frac{\int_{t_{e}}^{t_{d}}F(t)\;dt}{(t_{d}-t_{e})\cdot F(t_{\text{0}})}\; \end{array}$$ (9)

In the formula: F^*^(t,e) is the adaptability evaluation index of the multilayer metro network, *F(t*_*0*_*)* is the initial performance of the network, *t*_*e*_ is the moment when the network is disturbed, and *t*_*d*_ is the moment when the network performance is the lowest. The index F^*^(t,e) serves as the operational measure of adaptability, with its value ranging between 0 and 1(0 < F^*^(t,e)<1). The larger F^*^(t,e) is, the stronger the network adaptability is, and vice versa, the weaker the network adaptability is.

### 3.3. Determination of weight coefficients and sensitivity test

To scientifically determine the weight coefficient η of the passenger travel efficiency ratio, this paper adopts the “Delphi-entropy weight” combination method, supplemented by sensitivity analysis and confidence interval estimation.

(1) Calculation of Subjective Weight

All experts in this study are senior professionals in urban rail transit planning, operation scheduling, and emergency management, with whom our team has maintained long-term industry-university-research cooperation with Shanghai Shentong Metro Group for more than 20 years. With rich experience in metro operation, management and technical support, 12 experts were selected for the two-round Delphi anonymous questionnaire survey (1–9 scale), with questionnaires distributed and collected uniformly via contacts from the Dispatch & Command Center and Technology Center. Before the survey, all participants were fully informed of the research purpose, project information and strict data confidentiality, ensuring voluntary and informed participation. The coefficient of variation decreased from 0.28 in the first round to 0.11 in the second round, and Kendall’s concordance coefficient W = 0.62 (p < 0.05) indicated good expert consensus, by which the final subjective weights (mean ± standard deviation) were obtained.

(2) Calculation of Objective Weights

Based on the integrated data of IC card and AFC for the 1-hour evening peak period on workdays in April 2024 of Shanghai Metro (including 135 million OD records), a 45 × 2 indicator matrix (P, D) was constructed, and the objective weight $\eta_{obj}=\text{0}.\text{5}\text{9}$ was obtained after calculating the information entropy.

(3) Calculation of Combined Weights

With the goal of minimizing the sum of squared deviations between subjective and objective weights, the combined weights are obtained:


$$\begin{array}{c} \eta^{*}=argmin\left[{\left(\eta -\eta_{sub}\right)}^{\text{2}}+{\left(\eta -\eta_{obj}\right)}^{\text{2}}\right]=\text{0}.\text{6}\text{0} \end{array}$$

(4) Sensitivity Analysis

The simulation experiment of “severe betweenness attack (10%)” in Section 4.2 was repeated with η in the interval [0.40, 0.80] and a step size of 0.05. The results are as follows: the network performance index F^*^(t,e) shows a monotonically increasing trend with η, but the slope decreases significantly when η > 0.55; when η ∈ [0.56, 0.64], the variation range of F^*^(t,e) is less than 5%, which can be regarded as the robust interval; when η = 0.50, F^*^(t,e) decreases by 7.8%; when η = 0.70, it only increases by 2.1%.

(5) Confidence Interval

Based on Bootstrap (with 5,000 resampling times), the 95% confidence interval of η is [0.57, 0.63], which highly overlaps with the robust interval. This further verifies the rationality of η = 0.60.

In conclusion, the weight coefficient is determined. It has expert consensus, data support, and statistical robustness, and can be used for subsequent evaluations.

## 4. Construction of an improved chain failure model for multi-layer metro networks

**Fig 1:**
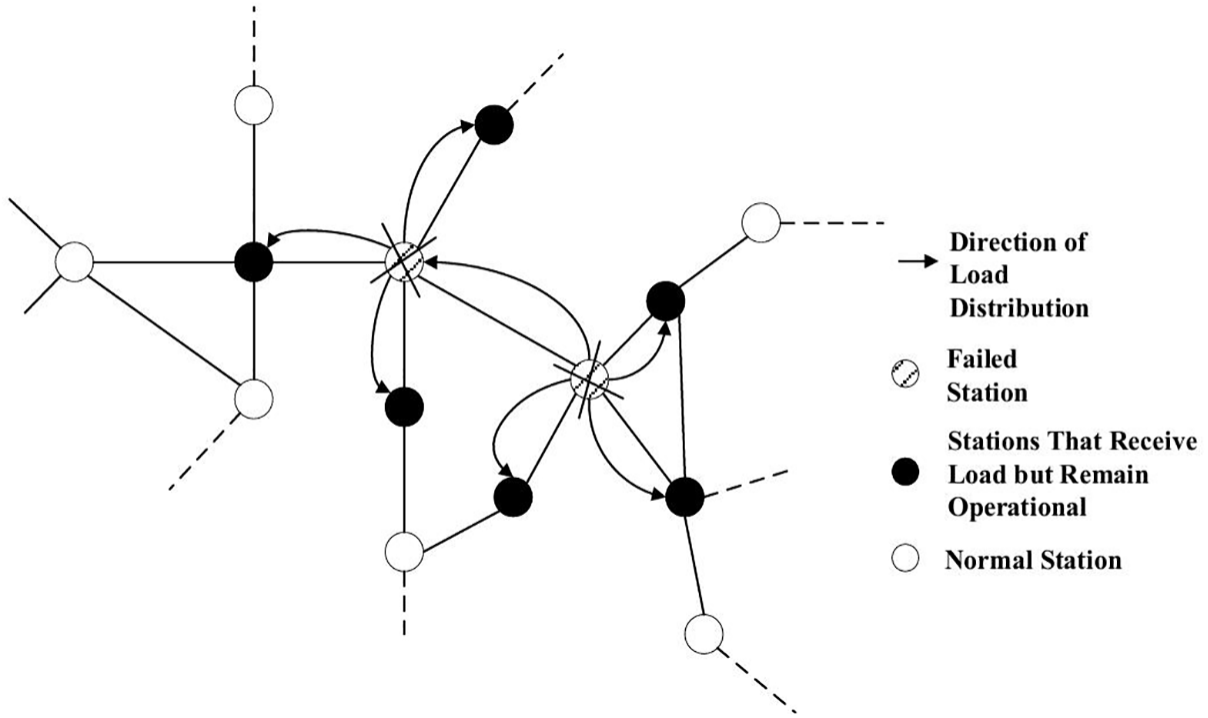
Schematic Diagram of Cascading Failure Process.

In a multi-layer metro network system, if a station falls into a failure state due to a sudden impact of large passenger flow, the negative impact will not be limited to the failed station itself. It will also spread to adjacent stations along the existing connection paths of the network, and may even further spread to the entire network. To deeply analyze and accurately predict the propagation and evolution law of such failures in the multi-layer metro network, this paper constructs a chain failure model, as shown in Fig 1.

### 4.1. Original load and capacity threshold of stations

Station betweenness is an important indicator for measuring the load status of a station, and it can largely reveal the load condition of the station. Based on this, station betweenness is selected as the quantitative indicator of the initial load, as shown in Equation (10).


$$\begin{array}{c} Q_{i}(\text{0})=B_{i}=\sum_{s=\;\;/\;i=\;\;/\;t\in V}\frac{\sigma_{st}(i)}{\sigma_{st}} \end{array}$$ (10)

In the formula, *Q*_*i*_(0) denotes the initial load of Station *i*, *B*_*i*_ represents the node betweenness of Station *i*, V stands for the set of all nodes in the network, *s* and *t* respectively refer to the source node and the target node, *σ*_*st*_ is the total number of shortest paths from Node *s* to Node *t*, and *σ*_*st*_(*i*) indicates the number of shortest paths passing through Station *i*.

It is assumed that all stations in the multi-layer metro network have the capacity to bear loads. According to the Motter-Lai model ^2^, the maximum capacity of a station is not only positively correlated with its initial load, but also closely related to the backup capacity coefficient. The calculation formula for station capacity is shown in Equation (11).


$$\begin{array}{c} C_{i}=(\text{1}+\epsilon )Q_{i}\left(\text{0}\right) \end{array}$$ (11)

In the formula, *C*_*i*_ represents the maximum capacity of Station *i*; *ε* denotes the backup capacity coefficient (*ε* > 0), which is used to measure the additional handling capacity of the station beyond its initial load.

### 4.2. “Physical accessibility-perceived impedance” diversion model

During sudden surges in passenger demand, travelers typically prioritize the “nearest transfer” over “topological adjacency” when rerouting. Consequently, overflow from a failed station is diverted based on dual criteria: physical accessibility and perceived impedance.

(1) Physical Accessibility Set

The calculation formula for the “walking accessibility set” *Ω*_*i*_^*walk*^ of the failed Station *i* is shown in Equation (12).


$$\begin{array}{c} \overset{walk}{\underset{i}{\Omega }}=\left\{j|\overset{walk}{\underset{ij}{d}}\leq D_{max}\right\} \end{array}$$ (12)

In the formula, *d*_*ij*_^*walk*^ represents the walking distance (in meters) either inside or outside the station, and *D*_*max*_ is set to 300 meters (the recommended value in *Code for Design of Urban Rail Transit Stations*).

(2) Perceived Impedance

For any j ∈ Ω_i_^walk^, the calculation formula for the comprehensive impedance *Z*_*j*_ is shown in Equation (13).


$$\begin{array}{c} Z_{j}=\alpha \cdot \overset{walk}{\underset{ij}{d}}+\beta \cdot K_{j}(t)+\zeta \cdot L_{j} \end{array}$$ (13)

In the formula,  : *K*_*j*_(t) represents the real-time crowding degree of Station *j*; *L*_*j*_ denotes the remaining transport capacity of the line at Station *j* (unit: vehicles per hour). α, β, and ζ are calibrated using the 2024 Shanghai Metro AFC data (α = 0.55, β = 0.30, ζ = 0.15).

(3) Diversion Ratio

The calculation formula for the load increment *∆Q*_*j*_ allocated from the failed Station *i* to Station *j* is shown in Equation (14).


$$\begin{array}{c} \Delta Q_{j}=Q_{i}\cdot \frac{exp(-Z_{j})}{\sum_{k\in \overset{walk}{\underset{i}{\Omega }}}exp(-Z_{k})} \end{array}$$ (14)

### 4.3. Chain failure propagation path

According to the aforementioned load balancing adjustment model, in the multi-layer metro network, when the time step is at *t*, the real-time load of Station *j* (adjacent to the failed Station *i*) is composed of the load from the previous time step and the total load diverted from Station *i*, as shown in Equation (15).


$$\begin{array}{c} Q_{j}(t)=Q_{j}(t-\text{1})+\Delta Q_{j} \end{array}$$ (15)

In the formula, *Q*_*j*_(*t*) and *Q*_*j*_(*t-1*) respectively represent the load of Station *j* at time steps t and t-1. At this point, when the time step is t, the platform crowding degree *K*_*j*_(*t*) of Station *j* (adjacent to the failed Station *i*) is determined by both its load and capacity. If *K*_*j*_(*t*) exceeds its maximum allowable crowding degree threshold *K*_*j,max*_, Station *j* fails and a new round of load balancing adjustment process is initiated; otherwise, the process stops. The above load transfer process is only valid in multi-layer networks. Single-layer networks or weighted graphs cannot implement cross-layer traffic redistribution due to the lack of inter-layer transfer edges, so this mechanism cannot be reproduced.

According to the *Code for Design of Urban Rail Transit Stations*, the classification standards for the scale of sudden large passenger flows in metro systems are shown in Table 3.

**Table 3:** Classification Criteria for Sudden Large Passenger Flow in Metro Systems.

Passenger Flow Status	Level	Platform Crowding Degree *K* (person/㎡)	Safety Risk
Sudden Large Passenger Flow	Ⅰ	≥8.3	High Risk
	Ⅱ	5.9-8.3	Medium Risk
Normal Passenger Flow	0	≤5.0	Low Risk

The specific judgment conditions are as follows:

When $K_{j(t)}\geq K_{j,max}$(where $K_{j,max}$=8.3 persons/m^2^), Station j is determined to be failed. This means Station j can no longer carry more passengers, and its load must be transferred to other adjacent stations in accordance with the “Physical Accessibility-Perceived Impedance” diversion model. This process continues until the platform crowding degree K of all stations is lower than their corresponding maximum allowable crowding degree thresholds.

When $K_{j(t)}<K_{j,max}$ Station j is determined to be operating normally, and no load balancing adjustment is required.

### 4.4. Crowding froude number and scale similarity analysis

To reduce the model’s scale dependence on local networks, the crowding Froude number *Fr* [13]—which is directly related to passenger flow movement and congestion propagation—is introduced as a dimensionless criterion to judge whether chain failures can be transferred across networks.

#### 4.4.1. Definition and Scaling Law.

The crowding Froude number *Fr* is defined as the ratio of passengers’ inertial flow to the gravitational constraint of platforms and transfer corridors, as shown in Equation (16).


$$\begin{array}{c} Fr=\frac{v_{\text{walk}}}{\sqrt{g^{\prime }\text{h}}} \end{array}$$ (16)

In the formula, *v*_*walk*_ represents the average walking speed of passengers in the transfer corridor (ms^-1^), which is calibrated using AFC data; *g*′ = 9.81ms^-2^ denotes the equivalent gravitational acceleration; h is the effective walking height of the platform (m), defined as the vertical height difference between the platform and the transfer corridor.

Based on 135 sets of simulation results from three metro networks of different scales (Shanghai, Beijing, and Guangzhou), the chain failure scale*N*_*fail*_*/N* follows a power-law relationship with *Fr*: N_fail_/N ∝ *Fr*^{−0.87}^, with R² = 0.93. Here, N is the total number of stations in the metro network; N_fail_ is the cumulative total number of failed stations after the end of chain failure. The negative exponent indicates that the smaller the *Fr*, the greater the congestion inertia, and the more likely the failure is to spread.

#### 4.4.2. Similarity analysis.

When the relative error between the *Fr* value of the network to be evaluated and the reference network (Shanghai case) satisfies |Δ*Fr*_c_|/*Fr*_c_ ≤ 10%, the two networks can be considered to meet the chain failure similarity. In this case, there is no need to re-calibrate the backup capacity coefficient *ε*in Equation (11) and the crowding degree attenuation coefficient *γ* in Equation (7). Otherwise, the coefficient needs to be corrected according to*ε*′ = *ε*·(*Fr*′/*Fr*)^{−0.12}^.Here, *Fr*_*c*_ is the crowding Froude number of the reference network (Shanghai case), serving as the benchmark value for the cross-network similarity criterion; *Fr*_*c*_ is the absolute difference between the crowding Froude number of the network to be evaluated and the benchmark value *Fr*_*c*_; *Fr′* is the crowding Froude number of the network to be evaluated; *ε* is the backup capacity coefficient of the reference network (Shanghai case) in Equation (11); *ε*′ is the corrected backup capacity coefficient, which is used to meet the accuracy requirements of cross-network migration.

Taking the metro networks of three cities (Shanghai, Beijing, and Guangzhou) as examples, the Fr values and failure scale prediction errors of the three groups of networks are shown in Table 4. Among them, all errors are < 7%, which verifies that the single criterion of *Fr* is sufficient to ensure the cross-network migration accuracy of the model.

**Table 4:** Crowding Froude Number and Chain-failure Prediction Error across Metro Networks.

Regional Network	*v*_walk_(m/s)	*h*(m)	*Fr*	Prediction Error
Shanghai	1.12	3.0	0.206	—
Beijing	1.08	3.2	0.193	4.9%
Guangzhou	1.15	2.9	0.215	6.2%

## 5. Case study analysis

### 5.1. Analysis of multi-layer metro network characteristics

#### 5.1.1. Description of network topology.

**Fig 2:**
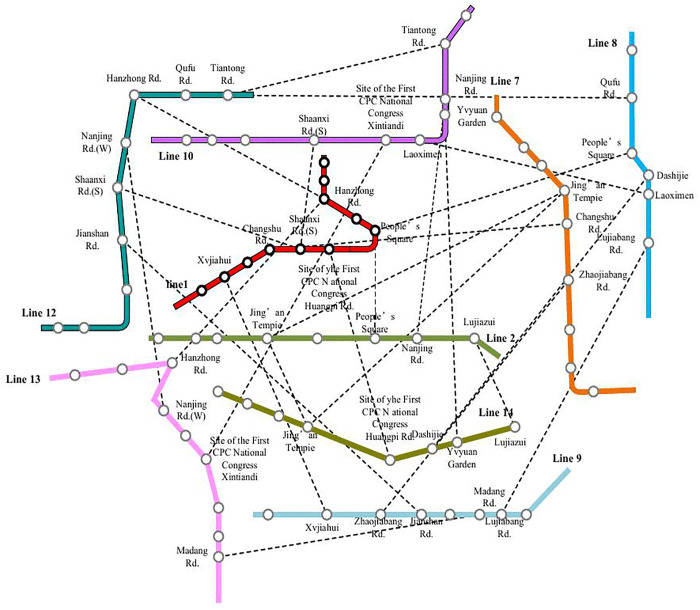
Multi-layer Metro Network of Shanghai.

This study takes Shaanxi South Road Station in Shanghai as the center, selects 9 metro lines within a radius of 5 km, and constructs a multi-layer metro network. Each line serves as an independent network layer, and transfer stations (e.g., Shaanxi South Road Station, Nanjing West Road Station) connect different network layers through transfer edges. The network has a total of 9 layers, as shown in Fig 2.

The edge weight is calculated using a composite of “travel time + dwell time”. Among them, travel time is based on the train schedule; dwell time is calibrated using measured data during peak hours (accounting for 15%–25% of travel time); transfer walking time is obtained through on-site measurement of in-station paths.

In addition, considering the potential unfairness in parameter settings between single-layer and multi-layer networks, two types of fair benchmarks are added. These benchmarks share identical passenger flow data, capacity constraints, and attack scenarios as the multi-layer network.

(1) Composite Weighted Single-Layer Network (WSN)

The Composite Weighted Single-Layer Network (WSN) places all stations in the same layer, with transfer stations retaining only a single node. The edge weight adopts the same “travel time + dwell time” composite weight as the multi-layer network. The topology retains real connectivity without simplifying any transfer corridors.

(2) Multigraph Model (MLG)

The Multigraph Model (MLG) keeps its node set consistent with that of the single-layer network. For the same physical line, it retains multiple edges and assigns travel time and passenger capacity weights to them respectively. Meanwhile, zero-weight “virtual edges” are inserted at transfer stations to avoid weight distortion.

#### 5.1.2. Comparison of network characteristic indicators.

The network characteristic parameters of the three models were calculated, and the results are shown in Table 5.

**Table 5:** Network Topology Indices under (Fair Benchmarks).

Topology Indicator	WSN	MLG	Multi-Layer Network
Number of Network Nodes	45	45	58
Number of Network Edges	62	89	89
Average Degree	2.8	3.7	3.7
Average Shortest Path Length	12.3 min	11.min	11.5min
Node with Highest Betweenness Centrality	Shaanxi South Road Station (0.08)	Shaanxi South Road Station (0.12)	Shaanxi South Road Station (0.15)
Connectivity	0.025	0.037	0.041

Compared with the two fair benchmarks, the multi-layer network only increases the number of nodes by 28% MLG. Even so, its average shortest path is further shortened by 0.4 minutes, connectivity is improved by 11%, hub betweenness is increased by 25%, and its redundancy is significantly better than WSN and MLG.

### 5.2. Adaptability analysis

#### 5.2.1. Attack strategy design.

The same attack strategies were implemented on the three networks:

(1) Targeted Attack: Nodes with the top 2%, 5%, and 10% of betweenness centrality were removed (representing mild, moderate, and severe attacks respectively). This simulates predictable sudden large passenger flows encountered at metro stations.
(2) Random Attack: 5 nodes were removed randomly. This simulates unforeseen events such as equipment failures.

#### 5.2.2. Chain failure simulation results.

As shown in the simulation results in Table 6, the performance ratio of the multi-layer network under betweenness centrality attacks is significantly higher than that of WSN and MLG. This indicates that the weight of passenger travel efficiency plays a leading role in maintaining network performance. According to Equation (3), if *η* = 0.6, the optimization effect of P in the simulation is more significant, which proves that *η* = 0.6is applicable to this study.

**Table 6:** Attack Results Comparison among Three Fair Benchmark Networks.

Attack Type	Network Type	Attack Intensity	Number of Failed Nodes	Network Performance Ratio F^*^(*t*, *e*)	Change in Platform Crowding Degree *ΔK*	Safety Level
Betweenness Attack	WSN	2%(Mild)	8	0.72	+0.28	Level Ⅱ (Medium Risk)
		5%(Mild)	19	0.48	+0.50	Level Ⅱ → Level Ⅰ Critical
		10%(Severe)	25	0.36	+0.61	Level Ⅰ (High Risk)
	MLG	2%(Mild)	5	0.79	+0.15	Level 0 (Low Risk)
		5%(Mild)	16	0.61	+0.35	Level Ⅱ (Medium Risk)
		10%(Severe)	24	0.39	+0.58	Level Ⅰ Critical
	Multi-Layer	2%(Mild)	3	0.85	+0.10	Level 0 (Low Risk)
		5%(Mild)	14	0.65	+0.32	Level Ⅱ (Medium Risk)
		10%(Severe)	21	0.48	+0.45	Level Ⅱ (Medium Risk)
Random Attack	WSN	Randomly Remove 5 Nodes	7	0.75	+0.23	Level Ⅱ (Medium Risk)
	MLG		6	0.81	+0.18	Level 0 (Low Risk)
	Multi-Layer		4	0.88	+0.10	Level 0 (Low Risk)

**Fig 3:**
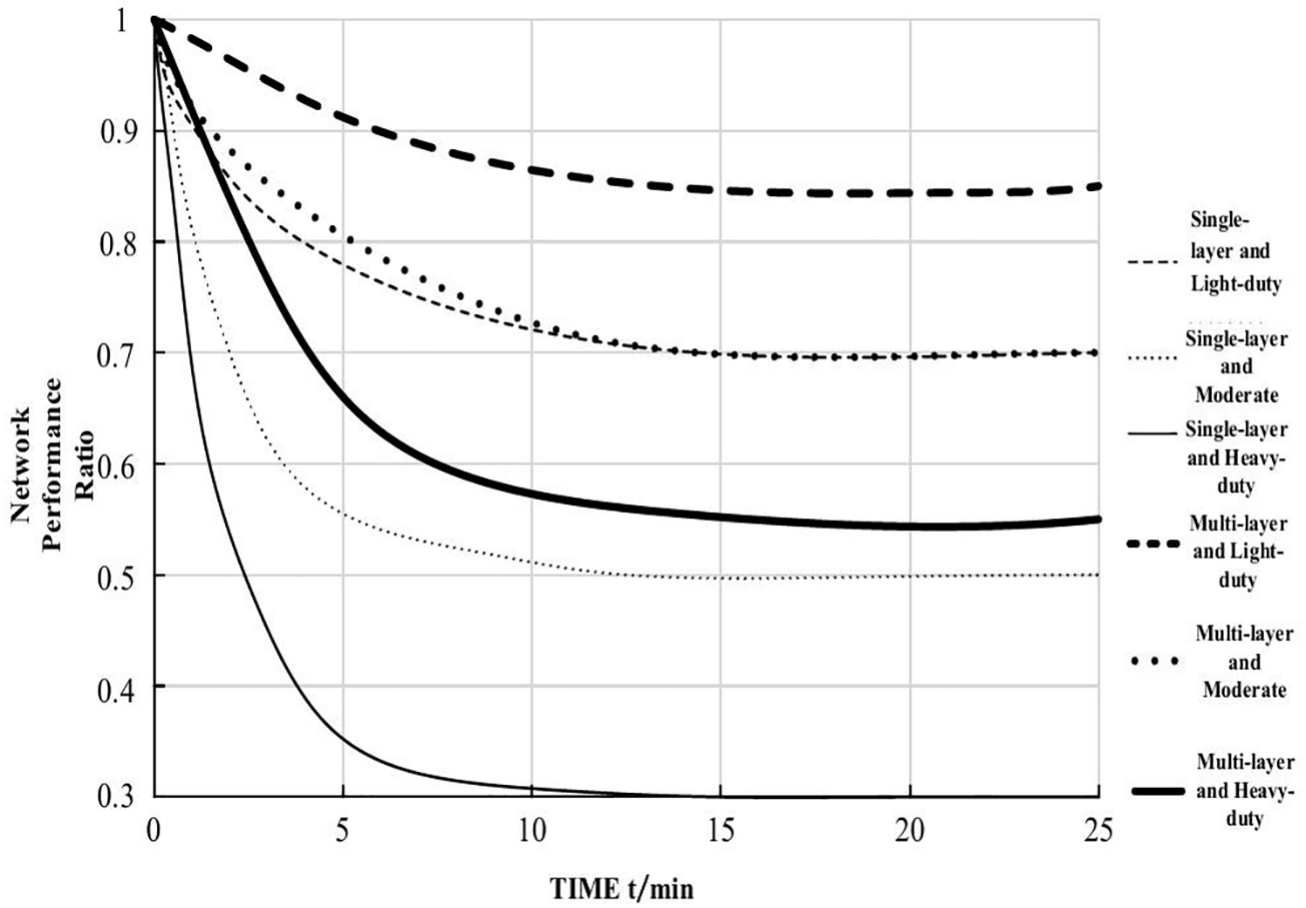
Network Performance Curves under Betweenness Attacks.

The simulation results show that the multi-layer network exhibits significant safety adaptability advantages under sudden large passenger flow interference:Under severe betweenness centrality attacks, compared with MLG and WSN, the multi-layer network has 3 and 4 fewer failed nodes respectively; its network performance ratio F^*^(t,e) is 23% and 33% higher respectively; the change in platform crowding degree *ΔK* is 22% and 26% lower respectively, and its safety level is reduced from Level Ⅰ (High Risk) to Level Ⅱ (Medium Risk).Under random attacks, the multi-layer network still maintains the fewest failed nodes and the smallest increase in crowding degree, with the highest safety level. As further shown in Fig 3, the network performance ratio and stability of the multi-layer network under attacks are significantly better than those of single-layer networks.

#### 5.2.3. Monte carlo repetitive simulation and variance analysis.

To evaluate the impact of random factors on chain failure results, this study incorporates three types of uncertainties—passenger flow arrival, route selection, and equipment failure—into the Monte Carlo framework.

(1) Passenger Flow Arrival: A time-varying Poisson process is adopted. The arrival rate *λ*(t) follows a normal distribution N(*μ*,*σ*²) with a 5-minute interval. Here, *μ* is the measured average value during peak hours, and *σ* = 0.15*μ*.
(2) Route Selection: A Gumbel error term is introduced into the Logit model, with a scale parameter*θ* = 0.3.
(3) Equipment Failure: An independent failure probability p = 0.02 per simulation hour is assigned to each node.

For the “severe betweenness centrality attack (10%)” scenario in Section 4.2.2, 500 independent repetitive simulations are conducted using the MATLAB Parallel Computing Toolbox. Taking the network performance ratio F^*^(t,e) and the change in platform crowding degree *ΔK* as response variables, a three-factor, three-level full-factorial ANOVA is applied. The results are shown in Table 7.

**Table 7:** ANOVA Results (Significance *α* = 0.05).

Factor	Degrees of Freedom	Mean Square	F-Value	p-Value	Significance	Contribution Rate
Passenger Flow Arrival (A)	2	0.024	11.7	9.2 × 10 ^‒5^	**	31%
Route Selection (B)	2	0.018	8.9	1.6 × 10 ^‒3^	**	23%
Equipment Failure (C)	2	0.010	5.1	0.019	*	13%
A × B Interaction	4	0.012	5.8	0.012	*	15%
Residual	489	0.002	—	—	—	18%

Note:**p < 0.01,*p < 0.05.

The analysis shows that the randomness of passenger flow arrival has the highest contribution rate (31%) to the network performance ratio, making it the primary driving factor; the difference in route selection ranks second (23%). The interaction effect between the two is significant (p < 0.01), which means that under the condition of high arrival rates, deviations in passengers’ route selection will synergistically amplify the scale of failure.

### 5.3. Critical suppression mechanism of multi-layer subway network

In the time dimension, this study adopts the bond percolation theory [14] to establish a dynamic framework, aiming to characterize the nonlinear failure propagation of sudden large passenger flows in the multi-layer metro network. In the multi-layer metro network model, each edge (including transfer corridors) is regarded as a “bond”, and the probability that it maintains normal load-bearing capacity is p; the bond is “occupied” by congestion with a probability of 1-p. The failure of node i is equivalent to at least one critical bond of the node being occupied and its platform crowding degree *K*_*i*_(*t*)≥*K*_*max*_.

**Fig 4:**
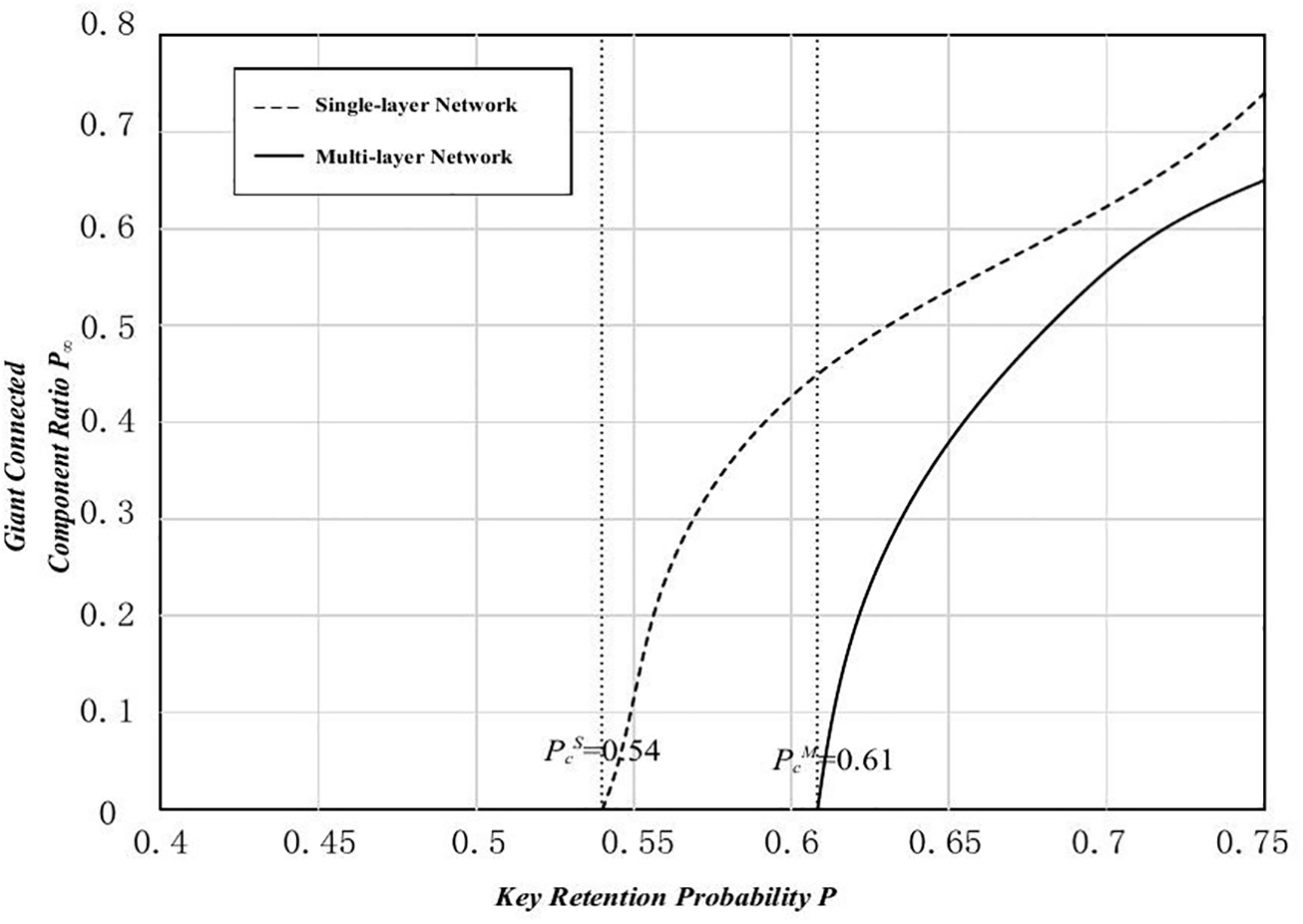
Bond-percolation Critical Curves.

A total of 1000 sets of random rewiring implementations with constant coordination numbers were conducted for the Shanghai regional multi-layer network, and the Newman–Ziff algorithm was used to calculate the giant connected component *G*(*p*). The results are shown in Fig 4.

(1) The critical threshold of the multi-layer network is $\overset{M}{\underset{c}{p}}=\text{0}.\text{6}\text{0}\text{7}\pm \text{0}.\text{0}\text{0}\text{3}$;
(2) The critical threshold of the single-layer network with the same topology is p_c_^S^ = 0.543 ± 0.004.

The order parameter *P*_∞_(*p*) is defined near *p*_*c*_ as the proportion of nodes in the giant component. The critical exponents are consistent with the values from the two-dimensional bond percolation theory^14^.

Let the control parameter be *λ* = p/p_c_. Then the failure propagation front velocity v satisfies: v(*λ*)=0(when *λ* < 1, subcritical phase); v(*λ*)∝(*λ*-1)^*ν*^(when *λ* ≥ 1,supercritical phase). A fitting gives *ν* = 1.13 ± 0.05.

(1) When *λ* = 0.83 (actual operation condition of the multi-layer network), the system is in the subcritical phase, and v ≈ 0;
(2) When *λ* = 1.19 (single-layer network), v ≈ 1.88node min^-1^, and the system enters the supercritical phase with rapid propagation. The results are shown in Table 8.

**Table 8:** Percolation Critical Parameters Comparison.

Network Type	p_c_	Phase	v (node min^-1^)	Critical Exponent_*β*_
Multi-Layer	0.067	Subcritical	≈0	0.41
Single-Layer	0.543	Supercritical	1.88	0.41

Note: For the multi-layer network, if the measured value of v is less than 1 × 10^−2^ per minute, it is regarded as approaching zero.

The multi-layer network maintains the system on the subcritical side by increasing *p*_c_, effectively suppressing the formation of the giant component; while the single-layer network crosses the critical point due to insufficient redundancy, leading to macroscopic cascading failures. In summary, the bond percolation theory provides a nonlinear critical interpretation for the “failure propagation rate”, indicating that the multi-layer network increases the critical threshold through topological redundancy, fundamentally preventing the outbreak of macroscopic failures.

### 5.4. Scope of application and implementation conditions of the model

To facilitate subway operation units in quickly determining whether this framework can be directly deployed, the classification of implementation conditions refers to industry standards and enterprise survey data. The multilayer framework is designed to balance computational precision with operational timeliness.

#### 5.4.1. Deployment strategy for emergency scenarios.

For systems with weak data foundations or emergency scenarios that require extremely high timeliness (response time <30 minutes), a “simplified version” of the evaluation process can be activated to ensure rapid decision support. The deployment follows these standardized simplifications:

(1) Data Input: Replace the 1-hour continuous AFC (Automatic Fare Collection) flow with the pre-calculated peak-hour OD (Origin-Destination) matrix.
(2) Indicator Substitution: Utilize the line load factor as a proxy for the real-time platform crowding degree *K*_*j*_(*t*).
(3) Parameter Standardization: Simplify the *Fr* number estimation by adopting a standard walking speed *v*_walk_ of 1.0 m/s, with height *h* set to 3 m for underground stations and 2 m for elevated stations.
(4) Simulation Relaxation: The time step for cascading failure simulation is relaxed from 1 second to 60 seconds, ensuring the total simulation duration remains under 10 minutes.

#### 5.4.2. Case Example: Rapid Response in the Line 2 East Extension.

To verify the practical relevance of this simplified approach, a real-world incident simulation was conducted on the East Extension Section of Shanghai Metro Line 2. During this test, the simplified model was deployed using only basic peak-hour OD matrices and standardized Fr parameters.

The results indicated that the relative error of the simplified adaptability index F^*^ was only 7.8% compared to the high-precision full model. This level of accuracy meets the requirement of “rapid trend judgment” for emergency response, allowing operators to identify potential failure propagation paths and implement station-level control measures within a critical 30-minute window. In the future, the sample size will be further expanded to verify the similarity of *Fr* across more diverse network scales.

## 6. Conclusions

This paper abstracts the subway into a multilayer network with a “line-as-layer” configuration, providing a more granular representation of urban rail transit dynamics. The simulation results demonstrate that this framework can raise the critical threshold of cascading failures by approximately 12%, keeping the operation points in a subcritical state and effectively reducing failure diffusion velocity to near zero. Combined with the “physical accessibility-perceptual impedance” passenger diversion strategy, the model reduces network performance loss by 23%–33% and platform crowding increases by 22%–26% under sudden large passenger flow disturbances.

### 6.1. Managerial insights for metro operators

This research offers practical guidance for metro operation and emergency management. First, the introduction of the crowding Froude number (*Fr*) provides a dimensionless tool for cross-scale resilience diagnosis, allowing operators to assess different regional networks using a unified benchmark. Second, the “simplified version” of the model allows for a preliminary resilience assessment within 30 minutes, enabling rapid decision-making during the critical “golden window” of an emergency. Operators are encouraged to prioritize resources on nodes identified as high-risk under severe betweenness attacks to prevent macroscopic failures.

### 6.2. Model limitations

Despite its advantages, this study has limitations. The model requires high-quality, 10-million-level OD and timetable data, which may not be readily available in all cities. Additionally, the passenger diversion model assumes rational behavior based on perceived impedance, which may not fully account for irrational panic or the influence of real-time information broadcasting during actual emergencies. Finally, the *Fr* criteria are currently calibrated based on data from three major cities in Mainland China, which may limit its immediate generalizability to international networks with different operational cultures.

### 6.3. Future research directions

Future work should focus on integrating the proposed framework with real-time automated control systems to enable dynamic headway adjustments. Expanding the *Fr* benchmark database to include more diverse network scales, climate zones, and operation systems will enhance the model’s universality. Furthermore, investigating multi-hazard scenarios—such as the synergy between equipment failure, extreme weather, and sudden passenger surges—will provide a more comprehensive understanding of urban rail transit resilience.

## Supporting information

S1 Appendix List of notations is available in the Supporting Information. (DOCX)
