# Supplementary material for: Study on the adaptability of multilayer subway network under sudden large passenger flow disturbances
Source: PLoS One. 2026 Jun 12;21(6):e0350567. doi: 10.1371/journal.pone.0350567 (PMC13262853; doi:10.1371/journal.pone.0350567)
Supplement: S1 Appendix — (DOCX) [file pone.0350567.s001.docx]

**Appendix: List of Notations**

To assist the reader, Table A1 provides a comprehensive list of the primary notations and variables used in the multilayer network model and the adaptability assessment framework.

**Table A1 Summary of mathematical symbols**

| Symbol | Definition |
| --- | --- |
| *G* | Collection of network layers |
| *C_αβ_* | Collection of transfer edges between layer *α* and layer *β* |
| *A*,*W* | Adjacency matrix and edge weight matrix, respectively |
| 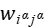 | Edge weight between nodes *i* and *j* within the same layer *α* |
| 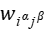 | Edge weight for transfer between nodes *i* in layer *α* and *j* in layer *β* |
| *F*(*t*) | Real-time network performance response function at time *t* |
| *F^*^*(*t,e*) | Cumulative adaptability evaluation index after disturbance *e* |
| *η* | Weight coefficient of the passenger travel efficiency ratio |
| *P*,*D* | Passenger travel efficiency ratio and network service efficiency ratio, respectively |
| *K_j_*(*t*) | Platform crowding degree of station *j* at time *t* |
| *C_i_* | Maximum passenger capacity of station *i* |
| *Fr* | Crowding Froude number for scale similarity analysis |
